# Inflammation balance in skeletal muscle damage and repair

**DOI:** 10.3389/fimmu.2023.1133355

**Published:** 2023-01-26

**Authors:** Huiyin Tu, Yu-Long Li

**Affiliations:** Department of Emergency Medicine, University of Nebraska Medical Center, Omaha, NE, United States

**Keywords:** complements, damage-associated molecular patterns (DAMP), immune cell, inflammation, sepsis, skeletal muscle

## Abstract

Responding to tissue injury, skeletal muscles undergo the tissue destruction and reconstruction accompanied with inflammation. The immune system recognizes the molecules released from or exposed on the damaged tissue. In the local minor tissue damage, tissue-resident macrophages sequester pro-inflammatory debris to prevent initiation of inflammation. In most cases of the skeletal muscle injury, however, a cascade of inflammation will be initiated through activation of local macrophages and mast cells and recruitment of immune cells from blood circulation to the injured site by recongnization of damage-associated molecular patterns (DAMPs) and activated complement system. During the inflammation, macrophages and neutrophils scavenge the tissue debris to release inflammatory cytokines and the latter stimulates myoblast fusion and vascularization to promote injured muscle repair. On the other hand, an abundance of released inflammatory cytokines and chemokines causes the profound hyper-inflammation and mobilization of immune cells to trigger a vicious cycle and lead to the cytokine storm. The cytokine storm results in the elevation of cytolytic and cytotoxic molecules and reactive oxygen species (ROS) in the damaged muscle to aggravates the tissue injury, including the healthy bystander tissue. Severe inflammation in the skeletal muscle can lead to rhabdomyolysis and cause sepsis-like systemic inflammation response syndrome (SIRS) and remote organ damage. Therefore, understanding more details on the involvement of inflammatory factors and immune cells in the skeletal muscle damage and repair can provide the new precise therapeutic strategies, including attenuation of the muscle damage and promotion of the muscle repair.

## 1 Introduction

Infection or tissue injury elicits a series of rapid innate immune responses required to eliminate infectious agents or damaged tissues, named as septic or sterile inflammation respectively. It is host defensive reaction to remove the invaders or clean damaged tissues for wound healing. However, uncontrolled inflammation possibly results in the tissue damage, even to a danger situation.

Responding to tissue injury, skeletal muscles undergo tissue destruction and reconstruction. According to the cellular and molecular events, there are five interrelated and time-dependent phases, including degeneration-necrosis, inflammation, regeneration, maturation/remodeling, and functional recovery (1). The sterile inflammation, as a result of trauma, typically occurs in the absence of any microorganism (2, 3). Similar to microbially induced inflammation, the sterile inflammation is marked by the recruitment of neutrophils and macrophages and the production of pro-inflammatory cytokines and chemokines, notably tumor necrosis factor (TNF) and interleukin-1 (IL-1). The inflammatory response could play central roles in bridging initial responses to muscle injury and timely muscle injury reparation (4) or triggering a vicious cycle to exaggerate the tissue damage (5, 6). Therefore, understanding the pathophysiological process of the sterile inflammation and controlling the sterile inflammation attack are very important for local tissue repair when the tissue is less regenerative capacity and prevention of remote organ damage.

## 2 Triggers of inflammation in the damaged skeletal muscle

### 2.1 Damage-associated molecular patterns promote inflammation

**Figure 1:**
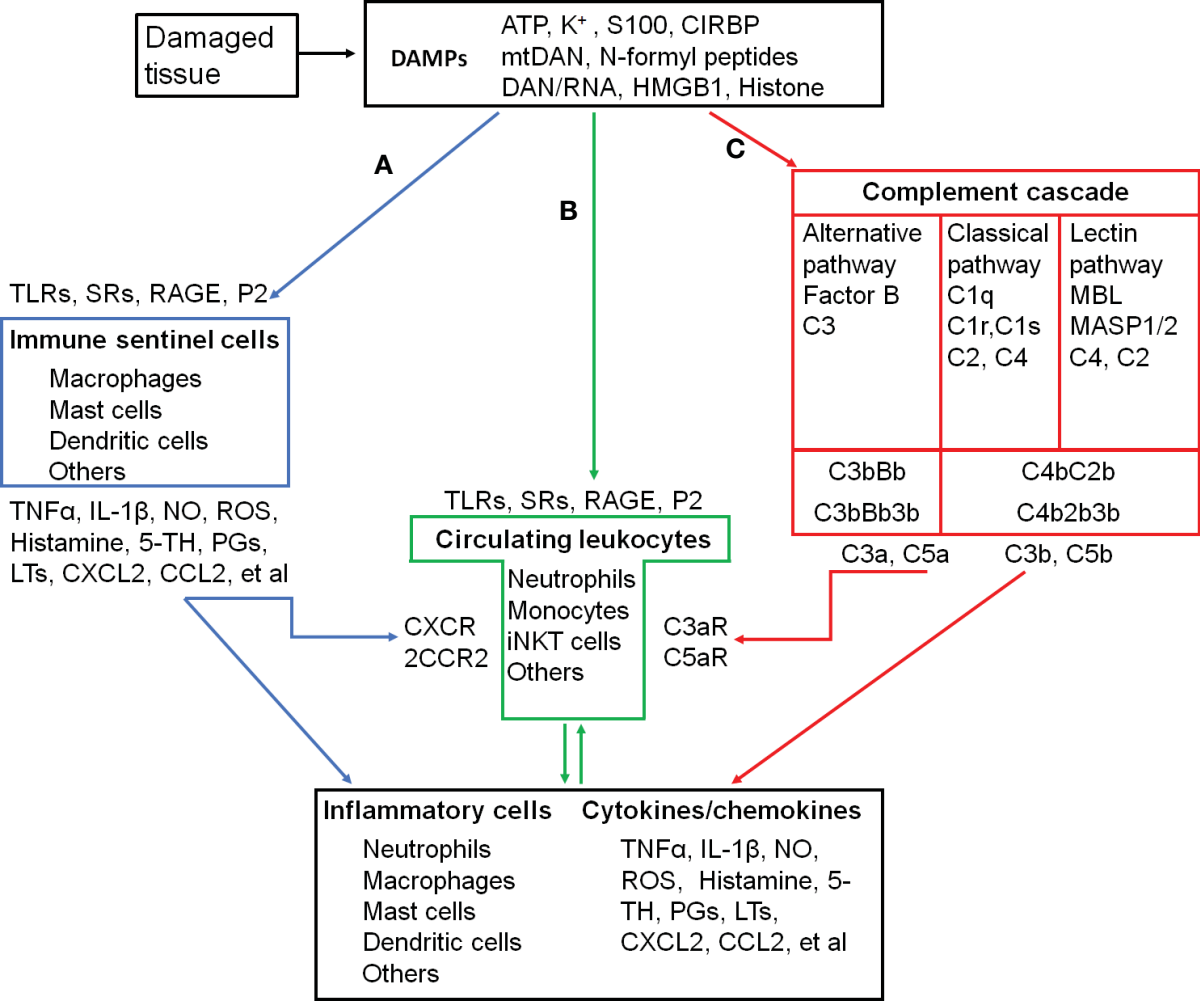
Inflammatory activation and its triggers in the skeletal muscle. **(A-C)**, three pathways that activate inflammatory cells. **(A)**, proteolytic cascades of complements are triggered by the damaged tissue and released C3a and C5a recruit and activate circulating leukocytes. **(B)**, DAMPs released into circulation are recongnized by circulating leukocytes, which are recruted to and activated in the injured site. **(C)**, damaged tissue-activated local immune sentinel cells release cytokines and chemokines to recruit and activate circulating leukocytes. ATP, Adenosine triphospate; CCL2, C-C motif chemokine ligand 2/Monocyte chemoattractant protein-1 (MCP-1); CCR2, C-C motif chemokine receptor; CIRBP, Cold-inducible RNA-binding protein; CXCL2, C-X-C motif chemokine ligand 2/macrophage inflammatory protein 2-alpha (MIP2-α); CXCR2, C-X-C motif chemokine receptor 2; DAMPs, Damage-associated molecular patterns; HMGB1, High mobility group box-1; IL-1β, interleukin-1β; iNKT cells, Invariant natural killer T cells. LTs, Leukotrienes; MBL, Mannose-binding lectin; MASP1/2, MBL-associated serine protease-1/2; mtDNA, mitochodrial DNA; NO, Nitric oxide; PGs, Prostaglandins; P2, Purinergic preceptors (P2Rs); S100, S100 protein; RAGE, Receptor for advanced glycation endproducts; ROS, Reactive oxygen species; SRs, Scavenger receptors; 5-TH, Serotonin; TLRs, Toll-like receptors; TNFα, Tumor necrosis factor α; C1 (2, 3, 4, 5), complement component 1 (2, 3, 4, 5); C1q, C1 complex componet– recognicition molecular C1q; C1r and C1s, C1 complex componet—tetrameric protease complex C1R_2_S_2_; C3bBb and C4b2b, C3 convertase; C2b, smaller fragment of C2 cleaved by C1s; C3a and C3b, two fragments of cleaved C3; C4b, Complement component C4b; C4bC2b, C3 convertase; C3bBb3b and C4b2b3b, C5 convertase; C5a and C5b, two fragments of cleaved C5; C3aR, C3a receptor; C5aR, C5a receptor; Factor B, Complement factor B; Bb, Fragment of complement factor B.

In addition to the exogenous signal that can be introduced into the body, the immune system can also sense danger molecules released from damaged or stressed tissues. Thus, the immune system can discriminate not only ‘self from non-self’ but also ‘healthy from damaged self’ (7). These danger molecules are intracellularly sequestered and are therefore hidden from recognition by the immune system under normal physiological conditions. They can be released in response to a variety of tissue trauma resulted from burns, cold, chemical insults, radiation, oxygen deprivation, nutrient depletion, auto-immune tissue destruction, tumors, and xenobiotics (7). An initial traumatic insult disrupts macrobarries such as the skin, and microbarriers such as cell membranes, which causes the release of multiple danger molecules. These endogenous danger molecules released from damaged or dying cells are termed as damage-associated molecular patterns (DAMPs), including high-mobility group box 1 (HMGB1), S100 proteins, heat shock proteins (HSPs), histones, mitochondrial DNA (mtDNA), and ATP (**Figure 1**) (6). They can be recognized by the innate immune system and are considered as key inducers of sterile inflammation following the tissue damage (8, 9).

During the cellular stress or injury, DAMPs can be released into the extracellular environment and blood circulation from damaged cells and are recognized by pattern recognition receptors (PRRs), such as Toll-like receptors (TLRs) and scavenger receptors (SRs), or non-PRRs, such as the receptor for advanced glycation end-products (RAGE) and purinergic receptors expressed on immune cells (8, 9) (**Figure 1**). After migrating through the vessel wall from the blood stream mediated by endothelial selectins and leukocyte integrins, leukocytes exit to interstitial space following a chemokine gradient where these chemokines are main ligands for C-X-C motif chemokine receptor 2 (CXCR2, a major chemokine receptor expressed in neutrophils and other immune cells) (8, 9). As the first recruited leukocytes, neutrophils are activated after their migration to the injury site along a gradient of DAMPs (8, 9). The proinflammatory monocytes, including monocytes with high expression of C-C chemokine receptor type 2 (CCR2^high^) and with low levels of C-X3-C motif chemokine receptor (CX3CR1^low^), successively transmigrate from blood stream in a CCR2-dependent manner, undergo *in situ* reprogramming into CCR2^low^ and CX3CR1^high^ alternative monocytes, and enter the injury site following the DAMP gradient. The *in-situ* reprogramming of monocytes depends on interleukin-4 (IL-4) and interleukin-10 (IL-10) produced by invariant natural killer T (iNKT) cells (9). The leukocyte recognition of DAMPs through PPRs or non-PPRs activates the downstream signaling through the adaptor proteins. For example, TLR2 and TLR4 on the leukocytes can be recognized by intracellular proteins HMGB1, HSPs, and histone released from the damaged tissue. They activate mitogen-activated protein kinases (MAPKs) and inhibitor of nuclear factor kappa B (IκB) kinase (IKK) to increase the production of the inflammatory cytokines from subsequently activated leukocytes through the activation of the transcription factors activator protein 1 (AP-1) and nuclear factor κB (NFκB), respectively (6, 8–11).

When the skeletal muscle damage occurs, the integrity of myofibers and other cells is severely compromised, and the plasmalemma permeability is alternated with uncontrolled ionic flux and the loss of a proper architecture (1). DAMPs released into the interstitial space and systemic circulation (12, 13) interact with PRRs or no-PRRs to promote inflammation (14). As pro-inflammatory mediators, specific DAMPs released from the skeletal muscle, including HMGB1 (15–17), ATP (18, 19), and mitochondrial DNA (20, 21), induce the secretion of pro-inflammatory cytokines and chemokines to trigger inflammation through TLR4/RAGE, P2X7R, and TLR9 on infiltrating/tissue-resident macrophages and neutrophils.

### 2.2 Complements promote inflammation

The complement is a system of more than 40 proteins in the plasma (soluble) and on cell surfaces (membrane-bound proteins). A number of complement proteins are proteases and widely distributed throughout body fluids and tissues without adverse effects. Activation of complements produces proinflammatory molecules, such as C3a and C5a to stimulate the inflammatory response (**Figure 1**) (22).

Complements are activated by three different recognition pathways (classical, alternative, and lectin), all of which lead to sequential enzyme activation, protein cleavage, and function-enabling protein conformational changes. Among these cascades, complement component 3 (C3) is the central molecule to the complement activation. These three pathways of the complement activation converge at the point of cleavage of C3 with generation of biologically active products, C3a and C3b.

The classical pathway is often referred to as antibody-dependent pathway because it is strongly initiated by binding complement component 1q (C1q) to the fragment crystallizable domain (Fc) of immunoglobulin M (IgM) or immunoglobulin G (IgG) clusters *via* the pattern recognition molecule (PRM) C1q subcomponent. However, C1q can activate complements by recognizing distinct structures on damaged cells directly or through endogenous substances, such as hyperphosphorylated tau (23, 24). C1q binding to damaged cells induces autoactivation of C1 complex to cleave complement component 4 (C4) and complement component 2 (C2) to form the C3 convertase (C4b2b).

Mannan-binding lectin (MBL) is a central recognition molecule in the lectin pathway. As the pattern recognition molecules binding to oligosaccharide structures on the surface of microorganisms, MBL, ficolins, and collectins assemble together and activate the MBL-associated serine proteases (MASP1/2). Once activated, MASP1/2 cleaves C4 and C2 to form the C3 cleaving enzyme-C4b2b. The lectin pathway is also triggered by released DAMPs, such as ATP (25) and cytoskeletal proteins (26), or unmasked sugars and neo-antigens (27) from damaged cells that can be recognized by and bind to MBL to initiates phagocytosis. The studies in MBL-deficient mice have demonstrated the impaired removal of damaged cells (26).

In the classical and lectin pathways, C3 convertase (C4b2b) sequentially cleaves multiple C3 proteins into C3a and C3b. Some of the C3b are associated with the C4b2b to form complement component 5 (C5) convertase (C4b2b3b) to cleave C5 into C5a and C5b (28).

The complement activation in the alternative pathway is initiated when spontaneously cleaved C3b directly attaches to a permissive/acceptor surface on the pathogen or damaged tissue (29, 30). Cleavage of inactive C3 protein can be spontaneously hydrolyzed into the functional fragments C3a and C3b at low level. Upon hydrolysis, the C3 protein undergoes a dramatic structural change that exposes a binding site for complement factor B to form C3bBb (a C3 cleaving enzyme complex) and C3bBb3b (an alternative C5 cleaving enzyme) (28).

Enzymatic cleavage of C5 to C5a initiates the terminal complement cascade, leading to polymerization of complement component 9 (C9) and insertion of membrane attack complex into cell membranes to lysis their targets. C3a and C5a, as potent proinflammatory mediators, recruit neutrophils, macrophages, mast cells, basophils, and lymphocytes to the injury site and promote inflammatory factor expression through C3a and C5a receptors in these immune cells (22, 31–33). C3b and C5b covalently attaches to pattern recognition molecules on the cell membrane to provide the opsonic signal to phagocytes for ingestion (24, 34, 35). During the physiological condition, complement activation with the low level of C3b deposition facilitates elimination of foreign and altered host cells (such as clearance of apoptotic cells) without the release of dangerous signals (32, 33, 36).

Skeletal muscles can produce complement components, include C1q, C1r, C1s, C2 and C4 (37). Complement activation is detected in damaged skeletal muscles from animals and human patients (38, 39). At the very early stage of skeletal muscle injury, the complement system is activated by its contacts with tissue intracellular components (40). The activated complements then recruit immune cells to cause inflammation. When complement activation is inhibited, the invasion of neutrophils and macrophages to the skeletal muscle is attenuated (41, 42) and muscle pathology is ameliorated (38).

### 2.3 Muscle-resident macrophages and mast cells promote inflammation

In the injury site, sentinel cells of the immune system (such as mast cells, macrophages, dendritic cells, innate lymphoid cells, and basophils) and non-immune system (such as endothelial cells) sense and react to DAMPs to produce proinflammatory cytokines (e.g., TNF-α and IL-1β), vasoactive amines (e.g., histamine and serotonin), nitric oxide (NO), ROS, neuropeptides, and arachidonic acid metabolites (e.g., prostaglandins and leukotrienes), which promote inflammatory responses through the recruitment of more neutrophiles and monocytes (**Figure 1**) (43–48).

Unlike monocyte-derived macrophages released from bone marrow and recruited to tissues during the injury with CCR2 activation (49), tissue-resident macrophages originate from the yolk sac and fetal liver during development and persist in many tissues *via* self-renewal. Tissue-resident macrophages express a wide array of receptors for the recogniztion of DAMPs, such as Toll-like receptors, nucleotide oligomerization domain (NOD)-like receptors, retinoic-acid inducible gene I (RIG-I) family, lectins, and scavenger receptors (50). Cells with these receptors act as local responders to the tissue damage and rapidly sense the death of individual cells. After initial recognition of the tissue damage, tissue-resident macrophages release inflammatory cytokines (TNF, IL-1, IL-6, IL-8, and IL-12) and chemokines (CXCL1, CXCL2, and CXCL5) (51) to drive the influx of inflammatory leukocytes, classically neutrophils and monocytes, from blood to the injured muscle (52).

Mast cells are located in the connective tissue that contacts close with the external environment. They are thought to play a pivotal role in allergy. IgE is thought to have a central role in the activation of mast cells through cross-linking of its high-affinity receptors (FcεIRs), whereas non-IgE-mediated activation of mast cells has been regarded as potentially important factor in the initation and amplification of acute inflammatory responses induced by tissue injury (53–55). DAMPs released from injured tissues, such as ATP (56) and IL-33 (45, 57), are recognized by mast cells *via* their receptors (P2X and P2Y receptors for ATP, ST2 receptor for IL-33), and then recognized DAMPs increase intracellular Ca^2+^ and activate mast cell degranulation. C3a and C5a, two complement components, can stimulate mast cell migration and degranulation *via* C3aRs and C5aRs (58, 59). The main contents in mast cell granules include histamine, heparin, serotonin, proteases, proteoglycans, cathepsin G, and cytokines (60, 61). Many of these mediators can induce inflammation and vasodilatation (55). The early disruption of the myofiber membrane elicits the accumulation and activation of muscle-resident mast cells. Activated mast cells subsequently degranulate and release inflammatory mediators (i.e. TNFα, IL-1, and histamine) to promote further immune cell recruitments (62, 63).

## 3 Inflammation promotes injured muscle regeneration

**Figure 2:**
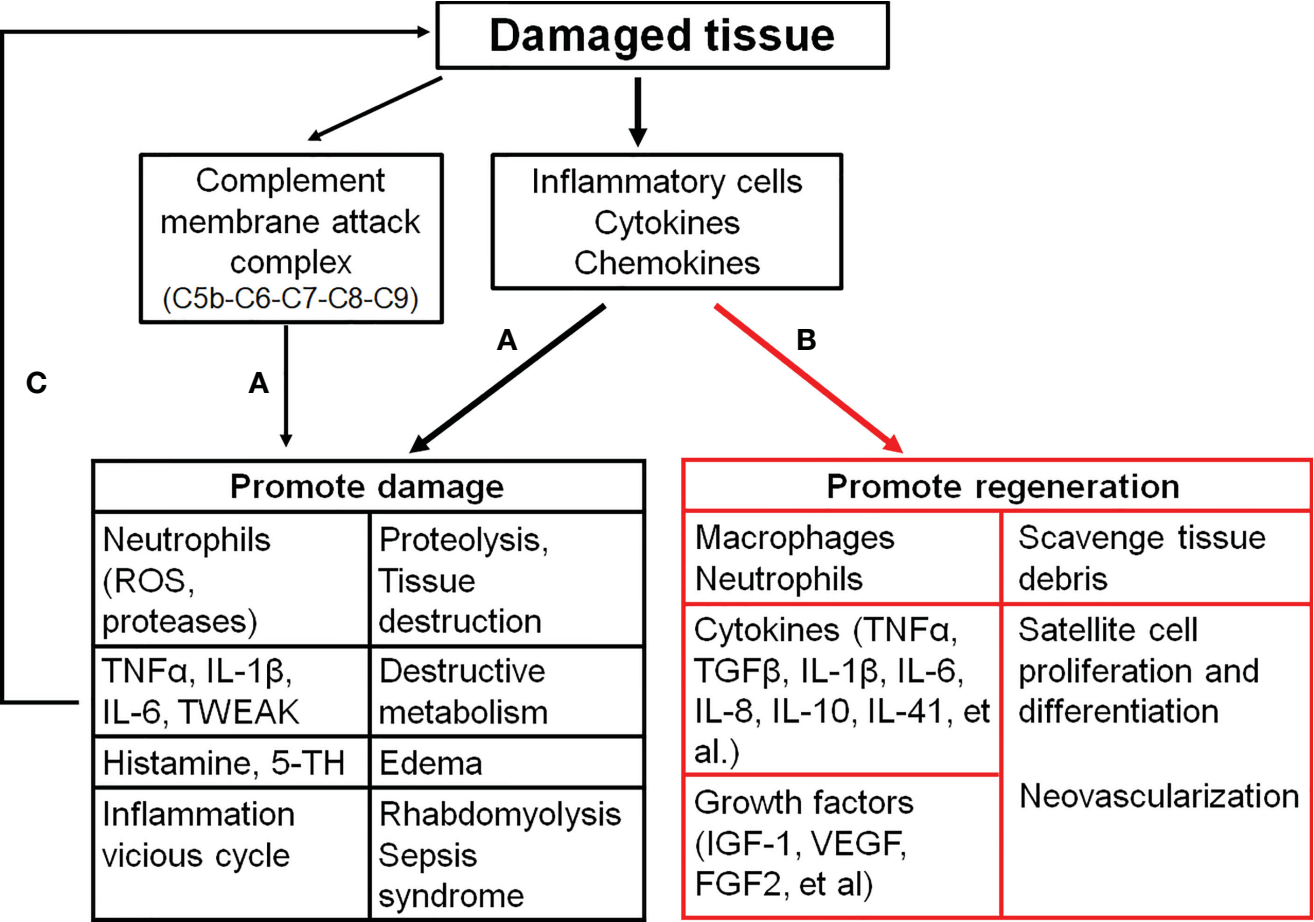
The effects of inflammatory activation on the skeletal muscle. **(A)**, inflammation distorys tissues; **(B)**, inflammation promtes muscle repair; **(C)**, a vicious cyle of the tissue damage and inflammation. C5b (6, 7, 8, 9), complement component 5b (6, 7, 8, 9); FGF2, Fibroblast growth factor 2; IGF-1, Insulin-like growth factor 1; IL-1β (6, 8, 10, 41), interleukin-1β (6, 8, 10, 41); ROS, Reactive oxygen species; TGFβ, Tranformaing growth factor-β; 5-TH, Serotonin; TNFα, Tumor necrosis factor α; TWEAK, TNF-like weak inducer of apoptosis; VEGF, Vascular endothelial growth factor.

The damaged skeletal muscle has the intrinsic capacity to regenerate and repair itself through myogenesis with the satellite stem cell activation triggered by damaged myofiber-derived factors (64). Activated satellite stem cells undergo proliferation and differentiation, which eventually fuse together or combine with damaged fibers to reconstitute the fiber integrity and function (65). Upon the tissue injury, infiltrating macrophages engulf and digest dead cells and cellular debris *via* phagocytosis, which causes a phenotypic change of macrophages to become healing macrophages for the regulation of inflammation, myoblast fusion and growth, fibrosis, vascularization, and final return to homeostasis (**Figure 2**) (66, 67).

### 3.1 Skeletal muscle damage triggers the inflammation to scavenge muscle debris

Necrotic myofibers may act as either atrophic factors to repress myoblast growth or physical barriers to prevent myoblast fusion. Engulfment of dead cells by phagocytes is a key event that ensures an efficient skeletal muscle regeneration to start the repair process and end the pro-inflammatory response (68). Most clearence of tissue debris is performed by macrophages and neutrophils through phagocytosis when damaged cells expose “find-me” and “eat-me” signals released from intracellular contents or appeared on their membranes (69, 70). These professional phagocytes are attracted by these specific “find-me” signals released by damaged cells. Then the multiple receptors on the cell membrane of phagocytes recognize phosphatidylserine (a key “eat-me” signal) exposed on the surface of damaged cells to capture damaged cells and the lysosomes in the phagocytes efficiently digest and decompose the internalized materials through phospholipases and hydrolases (71, 72).

As the professional phagocytes, macrophages perform their critical functions to scavenge debris for skeletal muscle repair. Several types of scavenger receptors are found in macrophages to bind with and internalize a variety of ligands, including endogenous proteins and pathogens, and modulate macrophage activation (73, 74). Macrophage scavenger receptor class A (SRA) binds with HMGB1 to eliminate HMGB1 from interstitial space by internalization, and then activates TLR4 to stimulate cytokine production (75). Additionally, elimination of exposed cellular components is a process to resolve inflammation (49, 68). The phagocytosis of muscle cell debris induces a switch from pro-inflammatory macrophages (M1) to anti-inflammatory macrophages (M2) (49, 68, 76) to stimulate myogenesis and fiber growth (77). Insufficient infiltration of macrophages or phagocytosis of necrotic fibers partially impairs myogenesis (78). Deletion of macrophage scavenger receptors decreases macrophage phagocytic activity on myoblast debris, and blocks the transition of macrophage phenotypes from M1 to M2, which delays muscle regeneration (79).

### 3.2 Inflammation-related cytokines promote muscle regeneration

Immune cells release a large number of cytokines, such as TNF-α, IL-1β, IL-6 and TGFβ, and growth factors. These cytokines can stimulate expansion of the muscle stem cells to promote repair (80).

TNF-α and IL-1β are mainly produced by macrophages. Both TNF-α and IL-1β directly activate the production of IL-6 in multiple cell types, including macrophages, T cells, and myofibers (81, 82). TNF-α and IL-1β induce the proliferation of cultured myoblast cells by similar mechanisms, whereas they regulate the transitory phase of myoblast differentiation through other mechanisms. Hight levels of TNF-α and IL-6 stimulate myoblast proliferation *via* STAT3 signaling, while they inhibit subsequent myoblast differentiation through NF-κB (p50/p65)-mediated degradation and destabilization of myogenic regulatory factors, including myoblast determination protein 1 (MyoD, a transcription factor that induces cell cycle arrest for the regulation of muscle cell differentiation) and myogenin (MyoG, a transcription factor that regulates myocyte fusion to induce myogenesis) (83). IL-1β decreases the level of myostatin, a negative regulator of muscle growth and regeneration to trigger myoblast proliferation (81). On the other hand, low level of TNF-α and IL-6 is necessary to facilitate later stages of myogenesis, because TNF-α and IL-6 at a low level stimulate myoblast differentiation and fusion through p38MAPK and the alternative NF-κB (p52/ReIB) pathway (81, 83). Ablation of TNF-α or IL-6 displays poor muscle regeneration (4).

Infiltrating macrophages recruited *via* CCR2 produce insulin-like growth factor-1 (IGF-I) in the injured muscle to stimulate muscle regeneration (84, 85). Meteorin-like protein (Metrnl/IL-41), identified as a myokine/cytokine, is secreted by the skeletal muscle (86) or activated macrophages (87). Metrnl/IL-41 promotes macrophage differentiation to an anti-inflammatory phenotype (M2) and induces IGF-1 production in M2 macrophages through activation of signal transducer and activator of transcription (STAT) proteins, which has a direct effect on the proliferation of primary muscle satellite cells (88). Macrophage-specific Metrnl/IL-41 knockout impairs muscle repair (88). M2 macrophages secret TGFβ to promote myogenesis through stimulating myogenic precursor cell commitment into differentiated myocytes and the formation of mature myotubes (89).

HMGB1 is also displays the regenerative character. These contrasting effects of HMGB1 depend on the redox state of cysteine residues (90). HMGB1 contains three cysteines (C23, C45, C106), which can be reduced or oxidized. If all cysteines are oxidized, HMGB1 has no known proinflammatory activity. The oxidation of the C23 and C45 residues leads to the formation of an intramolecular disulfide bond (dsHMGB1). Both dsHMGB1 and full reduced HMGB1 (frHMGB1) have the migrating function of macrophages. The dsHMGB1 is a proinflammatory cytokine to polarize macrophages toward pro-inflammatory phenotype (M1) through binding to RAGE/TLR4. The frHMGB1 induces distinct macrophages polarization phenotypes (90, 91). The frHMGB1 forms a heterocomplex with CXCL12 and activates CXCR4 expressed on stem cells to promote muscle regeneration and repair after acute muscle injury (92, 93). Compared to an HMGB1-RAGE/TLR4-axis in immune cells as a proinflammatory signaling pathway for the impairment of skeletal muscle function, the HMGB1-CXCL12-CXCR4 signaling pathway in stem cells promotes tissue regeneration in chronic inflammation diseases (16, 19, 93). The oxidation of HMGB1 cysteines can switch its extracellular activities from the orchestration of tissue regeneration to the exacerbation of inflammation. Pharmacological treatment with an engineered nonoxidizable variant of HMGB1 reduces inflammation and fibrosis, and improves muscle regeneration and functional performance (94). Additionally, studies with RAGE knockout or defective TLR4 demonstrate that HMGB1 binding to RAGE/TRL4 in the stem cells is also important to stimulate quiescent stem cell proliferation and differentiation, and further promotes muscle regeneration and neovascularization after the muscle is destroyed (15, 95–97). These systemically genetic modifications possibly impair the response of immunocytes to the tissue injury or other unknown signaling to reduce the cell proliferation and differentiation. TLR4 deficient mice developed into severe muscle injury (96), mild inflammation with low TNF-α and scarce macrophage infiltration, and poor muscle regeneration (98). RAGE is not expressed in the adult skeletal muscle, while it is transiently expressed in activated, proliferated, and differentiated satellite cells in injured muscles. The RAGE signaling represses Pax7 transcription in satellite cells through upregulation of MyoG, thereby accelerating muscle regeneration (myocyte fusion) and limiting satellite cell self-renewal (97). Satellite cells from RAGE knockout mice not only lack a high level of some cytokines (TNFα, MCP-1, IL- 6) in response to *in vivo* ischemia and *in vitro* stimuli with HMGB1 (99), but also exhibit the increase in basal satellite cells and delayed regeneration (myocyte fusion) of injured muscles (97).

### 3.3 Inflammation related cytokines promote neovascularization in skeletal muscle

Neo-angiogenesis is also necessary to establish a new vascular network for muscle repair. Both macrophages and mast cells contribute vascular regeneration (100, 101). In damaged skeletal muscles, endothelial-derived progenitors can contribute to neo-angiogenesis or fibrosis through the generation of mesenchymal/fibrogenic cells. The polarized macrophages affect the fate of endothelial progenitors during muscle regeneration after an acute injury. Experiments performed by Zordan et al. have demonstrated that the vast majority of endothelial-derived cells contributes to the formation of a new capillary network with macrophage infiltration (102). When circulating monocytes and infiltrating macrophages are depleted, angiogenesis and myogenesis are delayed with leading to a persistent fibrosis (102). Vascular endothelial growth factor (VEGF) signaling has a crucial role in this transformation. When the muscle trauma results in the disruption of blood flow and reduction of oxygen, hypoxia inducible factor (HIF) is elevated in the injured muscle, which induces the production and release of VEGF from macrophages to bind to VEGF receptors (VEGFR) expressed in endothelial cells for the proliferation, migration, and survival of endothelial cells (103, 104). Depletion of the macrophage recruitment reduces the VEGF production and impairs angiogenesis and skeletal muscle regeneration (102, 105). Macrophage-derived VEGF is also crucial to re-establishment of the neuromuscular junction (106). Other pro-angiogenic factors produced by macrophages, including fibroblast growth factor 2 (FGF2), IL-8, IGF-1, and IL-10 (101), also improve tissue repair. Additionally, macrophages stimulate myogenesis/angiogenesis coupling to orchestrate muscle regeneration through secreted osteopontin (107) and oncostatin M production (108).

Local mast cells are also associated with arteriogenesis and formation of collateral circulation in the skeletal muscle after ischemic injury (100, 109). In patients with peripheral arterial disease, or animal models with femoral artery ligation, mast cells are activated (62, 100, 109, 110). Activation of mast cells increases the proliferation of vascular endothelin cells and smooth muscle cells to promote neovascularization (100, 109). Treatment with cromolyn, a mast cell stabilizer, prevents the mast cell-induced arteriogenesis. Mast cells could directly contribute to vascular remodeling and vascular cell proliferation through the increase in matrix metalloproteinases’ (MMPs) activities and monocyte responses as well as supplement of growth-promoting factors, including VEGF FGF2, and platelet-derived growth factor BB (PDGF-BB) (109).

## 4 Inflammation aggravates muscle injury

As discussed above, inflammation is involved in skeletal muscle regeneration and has beneficial on muscle healing. However, a number of pro-inflammatory cytokines/chemokines also contribute to the pathogenesis of skeletal muscle injuries (**Figure 2**) (111). Therefore, anti-inflammatory modalities are commonly used for the treatment of various musculoskeletal injuries (112). Treatment with dexamethasone, a potent anti-inflammatory drug, protects the skeletal muscle from ischemia/reperfusion injuries through the inhibition of inflammation (113–117). Dexamethasone attenuates the alterations in microvascular function, edema, and necrosis of muscle fibers, and improves the muscle contractile function (113–117).

### 4.1 Inflammatory response aggravates muscle injury

After trauma, local and recruited immune cells are activated in the injured site. Activated lymphocytes, macrophages, and neutrophils contain radical forming enzymes in their intracellular granules to generate ROS. ROS can further increase tissue injuries and in turn enhance the immune responses to the tissue damage (118–120). Macrophages are rich in diverse growth factors and cytokines as well as ROS (121, 122). Therefore, macrophages play the opposite roles in the skeletal muscle to injure muscle cells or stimulate muscle regeneration. Pro-inflammatory cytokines released from activated phagocytes have been found to accelerate muscle protein degradation in patients with trauma (123, 124). Recently, Shang et al. reported that macrophages appear to compete with satellite cells for binding with glutamine to impede muscle regeneration (125). A macrophage-specific knockout of glutamate dehydrogenase inhibits the glutamine utilization in macrophages and improves earlier restoration of muscle functional capacity (80, 125).

Unlike macrophages, neutrophiles mainly release proteases to degrade cellular debris produced by the damaged tissue (126). As a part of neutrophil activation, neutrophils lead to proteolysis and removal of debris, high concentration of proteases, or other cytolytic and cytotoxic molecules released from neutrophils. These neutrophil-caused events can damage skeletal muscles and other healthy bystander tissues (48). Over-activation of neutrophils lyses the cell membrane (127) and results in the muscle damage (128). As one type of mediators, neutrophil-derived ROS are capable of direct lysis of the muscle membrane (129–131). Additionally, oxidative stress exacerbates the inflammatory responses and enhances the formation of fibrotic scar tissues after the skeletal muscle injury (132). Inhibition of the neutrophil infiltration attenuates the muscle damage (133, 134).

Mast Cells also play a prominent role in the ischemia/reperfusion-mediated cytotoxic injury in the skeletal muscle. Mast cell granules contain a number of mast cell-specific proteases, including tryptases, chymases, and mast cell carboxypeptidase A (MC-CPA) (135). These mast cell proteases are expressed at exceptionally high levels and kept in a fully active form. At the blood reperfusion, complement molecules, C3a and C5a, cause mast cell degranulation through activation of G-protein-coupled receptors (GPCR) on the mast cell surface. Additionally, increased ROS production also activates the intracellular signaling pathways to stimulate mast cell degranulation (60). When mast cells undergo degranulation, large amounts of enzymatically active proteases are thus released into the extracellular space to result in the tissue damage (136, 137). Drugs that target mast cells and their mediators (138), genetical deficiency in mast cells (138–140), or direct knockout of mast cell proteases (141) reduce the skeletal muscle ischemia-reperfusion injury accompanied with the attenuation of remote lung injury.

Since pro-inflammatory cytokines are found to accelerate the muscle protein degradation (142), lots of inflammatory mediators involved in the skeletal muscle injury have been reported (83, 141, 143–145). Although these cytokines modulate myofiber function and execute pleiotropic roles in the functional recovery of the skeletal muscle, they disrupt healing and exacerbate the muscle dysfunction when they form an aberrant downstream signaling pathway (21). Accompanied with the initiation of inflammation, HMGB1, amplifies the tissue damage and lethality through the HMGB1/RAGE axis (146). As a co-receptor of HMGB1 for the TLR activation, macrophage scavenger receptor A (SRA) mediates HMGB1 internalization (75) and interaction with TLR4 (147, 148) to exaggerate inflammatory responses. SRA-mediated influx of lipids through macrophage-modified lipoprotein uptake is thought to be involved in the formation of foam cells (149). In Duchenne muscular dystrophy (DMD), the inflammatory HMGB1-TLR4 axis promotes the dystrophic muscle pathological process and destroys dystrophic muscle fibers (16, 94). Ablation of TLR4 or inhibition of HMGB1 binding to TLR4 attenuates inflammation and improves the muscle histopathology and muscle force generation (16, 94).

TNF-α and IL-1β limit cell differentiation events and lead to muscle wasting (81, 150–152). Although high levels of TNF-α and IL-1β stimulate myoblast proliferation, they decrease the production of irisin, an important myokine that can stimulate myogenesis and muscle growth (81). Additionally, TNF-α inhibits myoblast differentiation through the degradation of myogenic regulatory factors (MyoD and MyoG) and downregulation of osteonectin, a secreted protein involved in the differentiation of pulp cells during the development and repair (81). TNF‐α blockade reduces TNF-α-associated tissue degradation and positively regulates the restauration of skeletal muscles upon injuries (153).

Complements not only activate inflammation but also aggravate the tissue injury (154). Complement activation converges at the point of cleavage of complement component 3 (C3) with the generation of biologically active products (C3a and C3b). C3a recruits neutrophils and macrophages to the injury site, while C3b activates the remainder of the complement cascade (C5-C9) to lead to the formation of membrane attack complex (MAC). MAC leads to the pore formation in the skeletal muscle membrane (38). Loss of the membrane integrity results in the release of intracellular contents, such as DAMPs. Then a severe and sudden reaction of the complement cascade against DAMPs could lead to the hyper-inflammation and tissue damage (155). Inactivation of complements or complement knockout reduces the invasion and activation of neutrophils and macrophages, and attenuates vascular damage (156, 157), muscle injury (38, 145, 158, 159) and edma (41, 159) in the damaged skeltal muscle. Additionally, inhibition of complements reduces remote pulmonary injuries secondary to tourniquet-induced skeletal muscle damage (157, 159).

### 4.2 The vicious cycle of tissue damage and inflammation-exaggerated muscle injury

After the muscle damage, intracellular DAMPs are released into interstitial space and the complement system and local macrophages/mast cells are activated. All of these local inflammatory events promote immune cell recruitment and activation with the high level production and release of inflammatory factors to trigger a vicious cycle of the tissue damage and inflammation (**Figure 2**) (5, 6).

Upon the tissue injury, released DAMPs activate the immune system to produce proinflammatory cytokines. As a specific DAMP and a necrotic marker for the immune system, HMGB1 initiates the proinflammatory signaling pathways and stimulates the immune cell activation (160). Activated immune cells also release HMGB1 (13, 161). Once the tissue damage becomes a prolonged event and the tissue repair fails, HMGB1 released by necrotic tissues and immune cells induces the second wave of inflammatory responses (162) or chronic inflammation (163, 164). Continuous inflammation can contribute to the development of various inflammatory diseases. Inflammatory diseases, in turn stimulate the secretion of DAMPs, thus establishing a vicious cycle of DAMPs production and inflammation (6, 162, 165). Continuously chronic inflammation results in muscle loss and atrophy (166).

In the alternative pathway of complement activation, C3 convertase initially cleaves C3 to C3a and C3b fragments. Then C3b binds with factor B to form C3bBb, a C3 convertase, to further cleave C3, which results in a positive feedback amplification loop (167) to produce a large amount of C3a and C5a fragments and promote the inflammatory responses, such as neutrophil recruitment and activation. When neutrophils are activated, they in turn activate the alternative complement pathway for the release of C5a fragments and the latter further amplifies neutrophil proinflammatory responses with another positive feedback loop (168). A severe reaction of the complement cascade can lead to the hyper-inflammation (30, 169) and tissue damage (29), or chronic inflammation (24).

Mast cell degranulation and macrophage activation can release proinflammatory cytokines (such as TNF-α, IL-1, histamine) (170, 171) to recruit more mast cells, neutrophiles and other immune cells. Then these recruited immune cells further release more proinflammatory cetokines (172). As the results, more immune cells infiltrate to the injured site following chemokine gradient to further promote inflammation, especially for neutrophil congregation in which a large number of neutrophils are recruited and collectively move toward the injured site in a very directed manner, i.e., prior activation of one or more ‘‘leading’’ neutrophils secretes leukotriene B4 (LTB4) and ATP. These molecules are recognized by A3 receptors (A3Rs) and LTB receptors (LTB4Rs) expressed on following neutrophils for the further neutrophil recruitment. This neutrophil–neutrophil signaling results in an autocorrelated behavior described as neutrophil swarming (9, 48, 173).

The muscle necrosis together with inflammation results in the accumulation of substantial amounts of fluid to raise the intracompartmental pressure in the affected limbs. The high level of the intracompartmental pressure provokes the additional damage and leads to more muscle necrosis, even limb amputation (174–177).

Not all responses to the muscle injury form a vicious cycle. Marcophages also suppress inflammation and autoimmunity in response to self-antigens caused during homeostasis (46). In the local minor injury, tissue-resident macrophages rapidly sense the death of individual cells and extend membrane processes that physiologically sequester pro-inflammatory debris to prevent initiation of the feedforward chemoattractant signaling cascade for the formation of neutrophil swarms (48). In the acute inflammation, tissue-resident macrophages release IL-10 to inhibit neutrophil scrolling and migration into the tissue and subsquently attenaute the inflammation (178).

## 5 Exaggerated inflammation causes remote organ damage

A local inflammation in the injured skeletal muscle can become more systemic and lead to the tissue damage and edema at distant sites (such as lungs). The decreased microperfusion of blood and tissue hypoxia due to the lung damage could cause more tissue damage (5).

### 5.1 Rhabdomyolysis

In the skeletal muscle, the damage initially occurs as “tears” in the membrane, which destroys the integrity of the sarcolemma and reduces the connection of the muscle membrane to cytoskeleton (179). Lack of the connection with cytoskeleton causes myofibers to be fragile and more sensitive to damage (180). This type of the damage can be exacerbated by the inflammatory response, leading to myofiber necrosis rather than repair (170).

Disintegration and necrosis of the skeletal muscle result in rapid breakdown of skeletal muscle fibers and release of muscular cell constituents into the extracellular fluid and circulation (12, 174), which is referred to as rhabdomyolysis. Rhabdomyolysis is usually caused by the direct muscle injury, such as trauma, and inflammation results in an additional injury and promotes rhabdomyolysis (181–183). Acute kidney injury (AKI) is one of the most severe complications after the occurrence of rhabdomyolysis and happens in 33-50% of patients with rhabdomyolysis (184) because of the toxicity of myoglobin to kidney tubular cells (182, 185). Previous studies also reported that severe acute inflammatory myosis without trauma triggers rhabdomyolysis with acute kidney injury (181, 186–188). These studies confirm that severe muscle inflammation contributes to the tissue damage and organ dysfunction (182). Another serious complication of rhabdomyolysis is severe hyperkalemia and the latter causes cardiac arrhythmia and arrest (189–191).

### 5.2 Severe inflammation in skeletal muscle results sepsis like syndrome

Thirty years ago, microbial pathogens were thought to cause the clinical sepsis syndrome and the relationship between the circulating mediators of inflammation and post-injury sepsis could not be imaged (192, 193). Now we understand that sepsis is fundamentally an inflammatory disease, and even infectious pathogens are not detectable in about one third of patients displayed clinical signs of sepsis (194), although sepsis is traditionally defined as life-threatening organ dysfunction caused by dysregulated host responses to infection (193). Sepsis is the consequence of exaggerated immune responses and widespread inflammation in the body to generate cytokine storm and results in life-threatening organ dysfunction (193, 195, 196).

Inflammatory cytokines are synthesized at the site of tissue injury where the sterile DAMPs released from wound sites activate innate immune cells (192). After severe tissue damage initiates massive activation of inflammatory mediators, the activated inflammatory mediators release into the bloodstream. Massive inflammatory mediators in the bloodstream result in systemic inflammation and multiple organ failure and death (12, 197), named sepsis-like systemic inflammation response syndrome (SIRS) (198).

In damaged skeletal muscles, proinflammatory cytokines, including IL-1β and TNFα, are significantly elevated (115, 116). As the early mediators of endotoxemia, IL-1β and TNFα mainly released by macrophages from the injured site into circulation cause septic shock and multiple organ injuries. Their antibodies have been used to prevent the organs against the lethal damage in mice suffered systemic inflammation (199–204). Additionally, IL-1β and TNFα stimulate the release of HMGB1, a late mediator of endotoxemia, and exaggerate the inflammatory damage (13). In a tourniquet-induced mouse hindlimb ischemia-reperfusion model, complement inhibition or neutrophil depletion attenuates remote organ injuries in the lung and liver (205), which confirms that local muscle injury results in the systemic inflammation and remote organ damage.

DAMPs are key inducers of systemic sterile inflammation. As a late mediator of endotoxin lethality, HMGB1 is secreted by activated monocytes and macrophages, or passively released from the damaged skeletal muscle into circulation (12, 13). In the human sepsis, the serum HMGB1 is increased, especially in non-survivors (13). Furubeppu, et al. have demonstrated that bilateral hindlimb ischemia not only induces severe muscle damage, but also significantly causes the elevation of serum HMGB1 levels and animal death (12). Treatment with anti-HMGB1 antibodies markedly improves animal survival (12, 13). Macrophage scavenger receptor A (SRA) mediates HMGB1 internalization (75) and interaction with TLR4 (147, 148) to enhance the development of the pro-inflammatory phenotype and mediate the morbidity and mortality of sepsis/septic shock, whereas the deletion of SRA or inhibition of SRA interaction with HMGB1 ameliorates sepsis/septic shock (147, 148).

Mitochondria and its components are another source for circulating DAMPs to activate systemic inflammation (192). The mitochondrial genome (mtDNA) contains CpG DNA repeats and also codes for formylated peptides. Unmethylated ‘CpG’ repeats existed in the mitochondrial DNA confer the affinity for innate immune cells with TLR9, and formylated peptides bind to formyl peptide receptor-1 to activate human polymorphonuclear neutrophils (PMN) through promoting Ca^2+^ influx and phosphorylation of mitogen-activated protein kinases (MAPKs), thus leading to PMN migration and degranulation (198). Intravenous injection of crude mitochondrial preparations causes neutrophil-mediated attack on the lung (198).

In addition to the involvement of neutrophil-mediated organ injury (198), the systemic inflammation also initiates clotting (206) to reduce blood flow into limbs and vital organs. Poor circulation leads to the organ failure and even animal death. Furthermore, clinical investigations and animal studies found that the systemic inflammation increases protein degradation and suppresses protein synthesis in the skeletal muscle, leading to an amplified net catabolism (143, 207).

## 6 Conclusion

**Figure 3:**
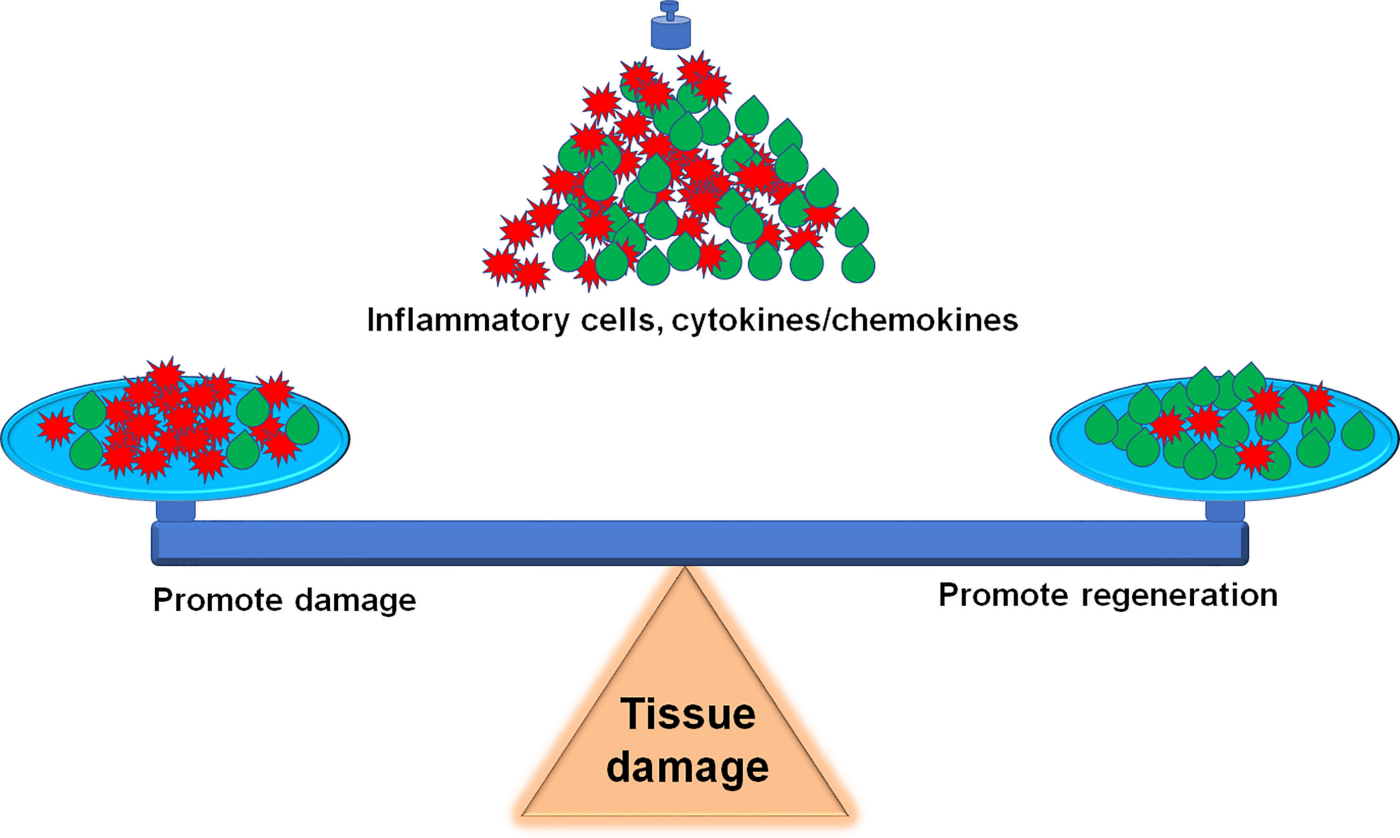
Inflammation balance in skeletal muscle damage and repair. How to accurately balance the beneficial and detrimental effect of inflammation during the skeletal muscle damage and repair is a challenge for precise therapeutic strategies.

Sterile inflammation is a host defensive reaction to scavenge damaged tissues for wound healing. The outcome is influenced by the magnitude of the inflammatory response whether the inflammatory process has an overall beneficial or detrimental effect on muscle function, and how to balance the beneficial and detrimental effect should be highlighted in the clinical practice (**Figure 3**). The inflammatory response consists of hormonal metabolic and immunological components and the extent correlates with the magnitude of the tissue injury (208). For tissue microlesions, tissue-resident macrophages sequester the damage through extending membrane processes to prevent initiation of inflammation (48). The macrophage activation can create a favorable microenvironment to release inflammatory cytokines for damaged tissue repair. This process is very useful for cells with the ability of regeneration. For other tissues without the regenerative capacity, inflammatory cytokines promote the fibrosis, such as in the heart. Given the skeletal muscle intrinsic capacity for regeneration and the benefit of inflammation on muscle repair, inflammation has less side effects on muscle recovery. However, inflammation also impairs muscle homeostasis in the patients with poor muscle stem cell pool, such as patient with peripheral arterial disease (209). Furthermore, severe tissue damage can cause systemic inflammation response syndrome and life-threatening organ dysfunction (12), especially, multiple remote organ damage, such as in the lung, heart, and kidney. To focus on this point, anti-inflammation could be a life-saving strategy. Sometimes, amputation rather than attempts at revascularization is the most prudent course to limit the toxic products from the damaged limb into the systemic circulation (210).

We updated the information about the inflammation balance in the skeletal muscle damage and repair in this review. It is a great challenge to balance the beneficial and detrimental effect of inflammation during the skeletal muscle damage and repair (**Figure 3**). Following the development of innovative techniques, including epigenetics, transcriptomics, single-cell RNA sequence (scRNA-seq) and proteomics, etc., more details on the involvement of inflammatory factors and immune cells in the skeletal muscle damage and repair can be further explored. For example, scRNA-seq analysis provides a new benchmark reference resource to examine the muscle tissue heterogeneity and identify potential targets for accurate therapy. Using scRNAseq analysis, Pang, et al. demonstrate that elevation of cell cycle genes in the specific monocyte/macrophages promotes inflammation and impairs skin wound healing (211). The use of scRNA-seq analysis resolves the cellular diversity of human muscles, including four types of stromal cells, five types of vascular cells, and two subpopulations of muscle stem cells (212). The proportion of different cell-types and cell-subtypes relates to age, sex, and the pathophysiology of muscle diseases (213). Krasniewski, et al. report eleven clusters of distinct macrophages in the mouse skeletal muscle, measured by scRNAseq analysis and also demonstrate that the enriched gene expression programs link to reparative, proinflammatory, phagocytic, proliferative, and senescence-associated functions (214). Therefore, the development of these new techniques will advance the new precise therapeutic strategies, including attenuation of the muscle damage and promotion of the muscle repair.

## Author contributions

HT and Y-LL contributed to drafting the work and revising it critically for important intellectual content. Both authors contributed to the article and approved the submitted version.
